# The importance of spatial heterogeneity and self-restraint on mutualism stability - a quantitative review

**DOI:** 10.1038/srep14826

**Published:** 2015-10-05

**Authors:** Rui-Wu Wang, Derek W. Dunn, Jun Luo, Jun-Zhou He, Lei Shi

**Affiliations:** 1 Center for Ecological and Environmental Sciences, Northwestern Polytechnical University, Xi’an, 710072, China; 2 Kunming Institute of Zoology, Chinese Academy of Science, Kunming, Yunnan, 650223, China; 3 Statistics and Mathematics College, Yunnan University of Finance and Economics, Kunming, Yunnan, 650221, China

## Abstract

Understanding the factors that enable mutualisms to evolve and to subsequently remain stable over time, is essential to fully understand patterns of global biodiversity and for evidence based conservation policy. Theoretically, spatial heterogeneity of mutualists, through increased likelihood of fidelity between cooperative partners in structured populations, and ‘self-restraint’ of symbionts, due to selection against high levels of virulence leading to short-term host overexploitation, will result in either a positive correlation between the reproductive success of both mutualists prior to the total exploitation of any host resource or no correlation after any host resource has been fully exploited. A quantitative review by meta-analysis on the results of 96 studies from 35 papers, showed no evidence of a significant fitness correlation between mutualists across a range of systems that captured much taxonomic diversity. However, when the data were split according to four categories of host: 1) cnidarian corals, 2) woody plants, 3) herbaceous plants, and 4) insects, a significantly positive effect in corals was revealed. The trends for the remaining three categories did not significantly differ to zero. Our results suggest that stability in mutualisms requires alternative processes, or mechanisms in addition to, spatial heterogeneity of hosts and/or ‘self-restraint’ of symbionts.

Mutualisms are reciprocally beneficial two-way inter-specific interactions^1^,^2^, and are characterized by a relatively large and predominantly sessile ‘host’ species interacting with larger numbers of individuals of a smaller, more mobile ‘symbiont’ species^3^,^4^,^5^,^6^. Mutualisms are globally widespread across diverse taxa and underpin much biodiversity^7^, with almost every species participating in at least one mutualism^8^. The benefits received by each mutualist usually involve inflicting a cost onto the other so the reproductive interests of both partners rarely align^7^. Natural selection should thus favour those individuals (usually symbionts) that obtain resources from hosts but fail to reciprocate (cheaters) or exploit the other mutualist (usually the host) at increasingly unsustainable rates, both of which will destabilise the mutualism^9^. Explaining why mutualisms are so widespread, and remain stable over evolutionary time, is thus a major scientific challenge^10^,^11^,^12^.

Stability in many mutualisms has been predicted theoretically to be affected by the spatial distribution of partners^13^,^14^,^15^,^16^,^17^. Where mutualist populations lack dispersal, host and symbiont interests are likely to become more closely aligned over evolutionary time^13^. For instance, hosts may be able to direct sanctions more effectively to symbionts that fail to reciprocate or provide reduced benefits^18^ in homogeneous populations, resulting in stronger selection for increased cooperation in symbionts^17^. Where mutualist populations are more heterogeneous and different host/symbiont genotype combinations are more likely to meet, hosts may be less likely to evolve mechanisms to reliably identify and then sanction individual symbiont cheats. Moreover, mutualisms that involve vertical symbiont transmission (host offspring are colonized with symbionts from a parent prior to dispersal) will likely effectively maintain associations between cooperative partners over successive generations^7^. Mutualisms characterized by horizontal symbiont transmission (new symbionts are recruited by hosts each host generation), may thus have reduced propensity to maintain stable cooperation between partners, similar to mechanisms operating in less structured populations.

At the spatial scale of the individual host, resource heterogeneity can increase the costs to those symbionts attempting over-exploitation. Analogous to selection against ever more virulent parasites and pathogens^19^, symbionts in some mutualisms have thus evolved ‘self-restraint’ in their exploitation of host resources^20^. For instance, in the fig tree-fig wasp nursery mutualism, in monoecious fig trees (*Ficus* spp.) fig flowers have evolved to be of highly variable lengths. Those long flowers that mature near the centre of the characteristic enclosed *Ficus* inflorescence (syconium or ‘fig’) tend to be galled by wasps, but shorter flowers near the inner fig wall are still pollinated by wasps but usually remain egg-free and become seeds. Various mechanisms increase costs to those wasps that attempt host resource saturation by laying their eggs in short flowers^21^ (e.g. reduced dispersal and increased parasitism rates of offspring^5^,^6^; barriers to efficient oviposition and/or offspring survival^22^). Stability is thus promoted indirectly by hosts presenting their wasp symbionts with a heterogeneous resource environment, that results in higher costs to ultra-exploitative individuals than the cooperative wasps that ‘allow’ their host to set seeds^23^.

Spatial heterogeneity between mutualists, and/or ‘self-restraint’ in symbionts, may lead to varying correlative effects between host and symbiont resource exploitation and hence reproductive success (Fig. 1). In mutualisms in which host populations vary spatially, those relatively most isolated from other populations are expected to exhibit a closer alignment of host-symbiont reproductive success than less isolated populations. A positive correlation between mutualist reproductive success is thus predicted to be stronger in populations in which mutualists are spatially close, and this will increase in strength and reach an asymptote over time as spatial heterogeneity is reduced, through the evolution of partner fidelity^24^ (Fig. 1). A consistently positive correlation between mutualist reproductive success may thus be indicative of spatial heterogenetity as a major contributory factor to system stability. However, prior to selection for close spatial associations between hosts and symbionts, a weak positive correlation between their reproductive successes, or no correlation, may occur.

In mutualisms in which symbionts have evolved ‘self-restraint’ and fail to fully exploit host resources, similar patterns between mutualist reproductive success can also be predicted (Fig. 1). For instance, in the fig tree-fig wasp mutualism wasp offspring and fig seed production may correlate positively due to more ovipositing wasps (foundresses) simultaneously increasing the overall levels of flower exploitation and the pollination of un-exploited flowers. However, when the potential for host over-exploitation is high, i.e when there are enough foundresses present to saturate all flowers with eggs, this is prevented by various mechanisms associated with variable flower morphology^5^,^25^. Instead, total wasp reproduction reaches an asymptote, with only a maximum of ~55–60% of the total flowers available being exploited by wasps^26^. When this asymptote is reached within a host population, host reproductive success will increase to a theoretical maximum but that of symbionts will remain relatively constant. No correlation between reproductive outputs of mutualists can thus generally be expected if ‘self restraint’ predominantly enables system stability, although a positive relationship will occur only when symbionts exploit their hosts to a level lower than any host imposed maximum threshold (Fig. 1). However, if the availability of any essential host resource becomes limited, for instance through total exploitation, in the absence of additional regulation of resource exploitation (e.g. effective host sanctions against cheats) the fitness of both mutualists will become negative, which may destabilise cooperation^12^,^27^,^28^,^29^. This is the well-known ‘tragedy-of-the-commons’^30^,^31^,^32^,^33^.

The diversity of mutualisms, and the fact that mutualists within each system are usually of widely different taxa^34^, presents problems to those attempting to use empirical data to quantify factors that promote mutualism in general^7^,^24^. To assess the potential contribution that spatial heterogeneity and ‘self-restraint’ may have in promoting stability in taxonomically distinct mutualisms, we thus used meta-analysis of the reported correlation between host and symbiont reproductive success in a variety of systems.

## Methods

### Literature search

We searched the ISI Web of Knowledge database using the keyword “mutualism.” Because three keywords “correlation coefficient”, “r” and “relationship” are all used to describe the relationship between mutualists, each of these terms was used in addition to “mutualism” in three separate searches. From the several hundred publications retrieved, we only selected those studies that reported the actual correlation coefficient (r) between the reproductive success of both mutualists in a particular system, and the sample size (N).

Many papers only presented the results of general and/or generalised linear models. We excluded these studies because most models included additional factors and/or covariates to explain the reproductive success of one mutualist, which makes the conversion of beta coefficients to correlation coefficients impractical. A total of 96 studies from 35 papers were included in the resulting meta-analysis (Appendix, Table A1).

### Data collection

Each separate experiment within each paper was treated as an independent ‘study’ for our database. For each study, we recorded the author, the time of publication, the symbiont, host, correlation coefficient between the reproductive output of each mutualist (r), and the sample size (N). We also categorised each host to one of four taxa: i) woody plant, ii) herbaceous plant, iii) insect, and iv) coral (Table A1).

Due to the variable biology of taxonomically different organisms the currency of reproductive success of mutualists varied among host types (as defined above), but within a particular host type, measures of reproductive success were more consistent. For instance, for insects (as hosts or symbionts) the numbers of larvae or adult offspring produced were often presented; for woody or herbaceous plants, seed production was a common, straightforward measure of reproductive success and we included papers that presented these data. We did not include papers that presented only measures such as plant biomass because this does not directly relate to reproduction. Likewise, for legumes-rhizobia (that contributed to data for herbaceous plants), we included papers that presented seed production of the plants and rhizobial node numbers but not those that only presented measures such as plant and node biomass. For coral-zooxanthellae mutualisms we included papers that presented data for gamete and/or larval production.

### Statistical analyses

All analyses were performed using a combination of Metawin version 2.0 and Matlab 7.10 software. The correlation coefficient r was treated as the effect size. Before each analysis, we first made Fisher’s *z* correlation coefficient transformation^35^,^36^.

After examining the potential of publication bias with funnel plots of the effect size by producing a Normal Quantile Plot^37^, we identified three data points (numbers 53, 54 and 55) with high residual values. These outliers were removed from the analyses. The final data set is based on 93 studies, which when represented by a funnel plot of sample and effect sizes, is approximately symmetrical (Fig. 2). For all four categories, the ratio of squared pooled variance (0.550) to mean study variance (0.057) is 9.584, which suggests that a test for homogeneity of effect size based on a random (or mixed) effects ANOVA is appropriate for these data^38^. Tests for homogeneity of effect size were based on Q statistics, with larger values indicating greater heterogeneity in effect sizes among comparisons. Total heterogeneity (Q_T_) can be partitioned into within-group heterogeneity (Q_W_) and between-group heterogeneity (Q_B_), which is analogous to the partitioning of variance in an ANOVA with multiple factors.

Total heterogeneity (Q_T_ = 157.11, df = 92, *P* < 0.001) was significant, with an overall 95% bootstrap confidence interval (95%BCI) ranging from 0.072 to 0.376. Comparison among groups revealed significant heterogeneity in effect size (Q_B_ = 22.91, df = 3, *P* < 0.001; Fig. 3), which reflected a relatively high positive value for corals (comparison of corals vs non-corals: Q_B_ = 21.29, df = 1, *P* < 0.001; Fig. 3). We found no evidence of heterogeneity in effect size between mutualisms involving woody plants, insects, and herbaceous plants as host species (Q_w_ = 0.93, df = 2, *P* = 0.63).

Ninety-five percent bootstrap confidence intervals (95% BCI) around the mean effect size were calculated in the framework of a random effects model for each host category (the 95% confidence interval and bias confidence interval had similar patterns). An effect size is only statistically significant if its confidence intervals do not overlap with zero^39^. For mutualisms with woody plant hosts (E = 0.10, 95% BCI = −0.126 to 0.429), insect hosts (E = 0.107, 95% BCI = −0.184 to 0.393) and herbaceous plant hosts (E = 0.239, 95% BCI = −0.028 to 0.493), all three confidence intervals overlap with zero, and are thus not significantly different to zero (Fig. 3). Only for mutualisms involving coral hosts (E = 1.22, 95% BCI = 0.547 to 1.68) did both confidence intervals exceed and differ significantly to zero (Q_T_ = 157.11, df = 92, *P* < 0.001, overall 95% BCI = 0.090 to 0.431; Fig. 3).

## Discussion

**Figure 1:**
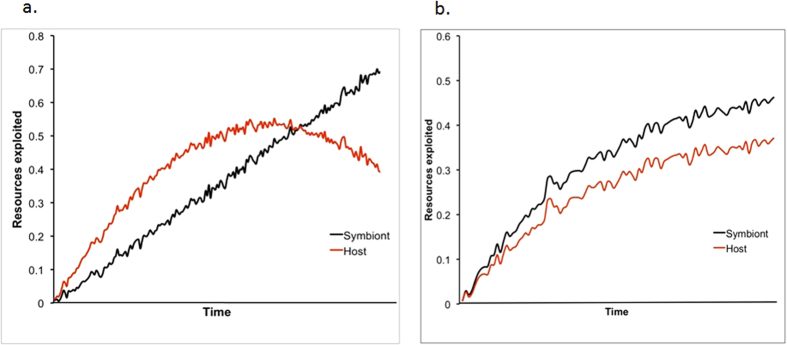
The degree to which each mutualist (red line = symbiont; blue line = host) utilizes the resources provided by the other mutualist over time. An increase in resource usage is assumed to translate directly to an increase in the fitness of the exploiting mutualist. (**a**) In systems that lack spatial heterogeneity, the fitness of both symbionts and hosts will increase bilaterally before any common resource becomes exhausted. However, the fitness increase of symbionts will be at the expense of host fitness at the point resources are fully exploited. (**b**) In systems in which spatial heterogeneity occurs, the fitness of both hosts and symbionts will each increase until an asymptote is achieved and the system will become stable.

**Figure 2:**
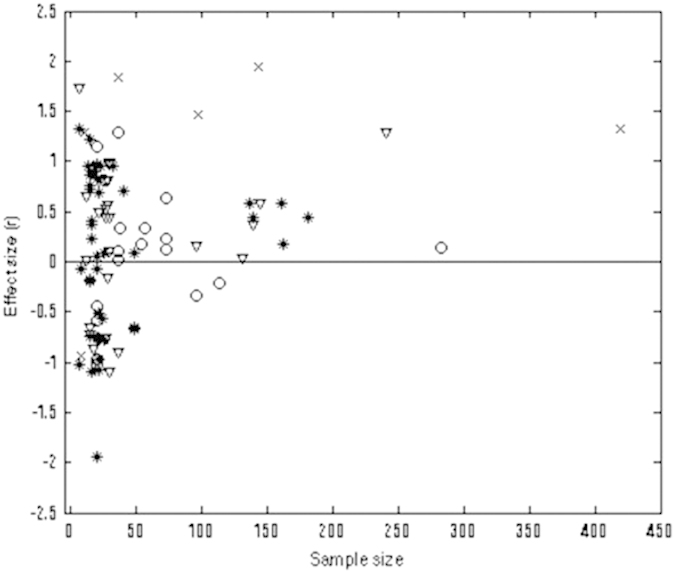
Funnel plot of the effect size, split by host type: Woody plant (*), Insect (o), Herbaceous plant (∇), and Coral (×).

**Figure 3:**
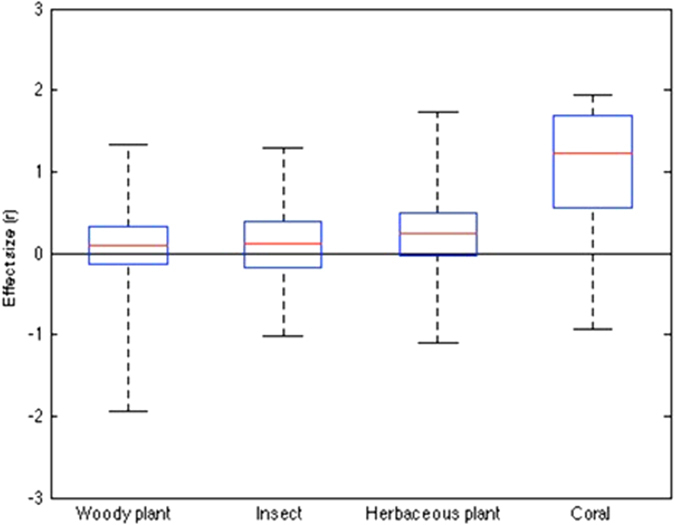
Mean effect size of hosts of the four categories of mutualism used in the analysis.

Overall, we found no overall significant trend associated with the strength and direction of the correlation between mutualist reproduction. However, there was significant heterogeneity between mutualisms of the four different categories of host used for the study. For cnidarian corals, we found that the correlation between host coral and zooxanthellae symbiont reproduction was significantly positive. In the remaining three groups of mutualisms with either woody plants, insects or herbaceous plants as hosts, no significant trends were apparent. These results suggest that spatial heterogeneity of mutualists and/or ‘self-restraint’ of symbiont virulence may not generally promote stability across mutualisms of these four host types. Although a zero correlation between mutualist fitness can be sometimes present when either and/or both mechanisms are operating (Fig. 1), an overall positive correlation is most likely (Fig. 1).

The strength and direction of the correlation between partner fitness has long been recognized as an important predictor of the nature of ecological interactions^40^,^41^. In general, a positive correlation is suggestive of an alignment of reproductive outputs between individuals, and when this reflects inter-specific processes, indicates a mutualistic relationship^1^. A negative relationship is indicative of parasitism, by which one partner gains by reducing the fitness of the other. The lack of a significant correlation between the fitnesses of two interacting species over protracted time periods can be interpreted as commensalism—each partner’s reproductive success is unaffected by the other partner^42^. However, this can be the case when two species initially become associated with each other, or when hosts prevent symbionts exploiting the benefits they provide beyond a certain threshold (Fig. 1). Most of the systems involved in our study are obligate, i.e. each partner requires the benefits provided by the other in order to reproduce. Why then was a positive correlation not the general trend among the diverse mutualisms involved in our study?

First, a positive fitness relationship between mutualists may not be consistently present, even in systems that are clearly stable over evolutionary time. In other words, mutualisms may not be in equilibrium and may fluctuate in space and over time, because variable ecological factors can alter the costs and benefits of cooperation to either mutualist. For example, in at least one fig tree-fig wasp mutualism cooler seasonal temperatures increase the lifespans of symbionts (pollinating wasps), thus increasing their potential to exploit host resources (laying eggs in more fig flowers)^20^. Although in warmer months there is a positive relationship in this system between mutualist reproductive success (wasps do not live long enough to fully exploit hosts, i.e. there is ‘self restraint’ in symbionts), the correlation becomes negative in the cooler winter^20^,^21^. A fluctuating correlation between mutualist reproductive success^21^,^43^, may thus result overall in a zero net effect depending on when particular systems were sampled which may have been reflected in some of the studies used for our analyses.

Second, publication bias may have affected the trends we revealed. Publication bias usually results from studies reporting non-significant effects having reduced likelihood of publication^37^,^44^, for example due to editorial policies favouring significant results or if studies reporting non-significant results are less likely to be submitted for publication, i.e. the ‘file drawer’ effect^45^. If the trends we found in our analysis were affected by publication bias, any bias would be centred on studies involving cnidarian corals and their zooxanthellae symbionts. Similarly, the narrow criteria by which we selected papers for inclusion in our meta-analysis may have resulted in reduced statistical power that increased the likelihood of type II errors. Because positive and negative correlations between mutualist reproductive success were reported fairly evenly among the papers we examined (Fig. 1; Table A1; see also methods section), we obtained a statistically insignificant result from the procedure we used to detect publication bias, suggesting a taxon specific bias is unlikely. Furthermore, our bootstrapping methods would have countered any potential problem of reduced statistical power adversely affecting the results^46^. However, a recent taxon specific quantitative review found a positive correlation between legume-rhizobial fitness^47^. This legume-rhizobium study used wider criteria for inclusion in a meta-analysis (partly due to more factors that equate to host or symbiont fitness) than did ours, and the results contradict to a certain extent our result for herbaceous plants. Our data for herbaceous plants included studies that had taxonomically different symbionts (e.g. insects, bacteria), which may have also contributed to an overall nil effect if systems with taxonomically similar hosts with taxonomically different symbionts had opposing effects. We suggest a future meta-analysis of mutualisms involving herbaceous plant hosts, that uses wide inclusion criteria due to taxonomically varied symbionts with different reproductive biology, will be informative.

The trend we identified between cnidarian coral hosts and their zooxanthellae symbionts, is suggestive of biological factors within these systems resulting in consistently positive correlations between the reproductive success of both mutualists. In other words, there may generally be less conflict in cnidarian coral-zooxanthellae mutualisms than the other systems involved in our meta-analysis. Vertical transmission of symbionts, whereby host offspring inherit a small proportion of their maternal symbiont population^2^,^48^, is predicted to facilitate cooperation between hosts and symbionts because it limits the opportunities for ‘cheats’ to invade symbiont populations^2^,^7^. For instance, the close spatial association with symbionts gives hosts greater opportunities to increase the costs to symbiont ‘cheats’ by deploying sanctions. However, although some cnidarian corals that produce brooded offspring vertically transmit symbionts between generations, in general there is horizontal symbiont transmission^48^; most corals are broadcast producers of offspring that acquire their symbionts from the wider environment^7^,^48^. Moreover, some of the additional host taxa groups in which we detected no overall correlation between host-symbiont reproductive success, there is generally vertical transmission of symbionts, for example between aphids and their bacterial endosymbionts *Buchnera aphidicola*^2^. This suggests that spatial heterogeneity alone may not be as important a factor in producing a strong overall close association between host-symbiont reproductive success as other processes.

Cooperation between coral-zooxanthellae mutualists regularly breaks down^48^, which appears contradictory to our finding that the reproductive outputs of both mutualists is generally positively correlated in these systems. When cooperation breaks down corals become devoid of their symbionts, which results in the well-known phenomenon of coral bleaching. This is generally thought to be due to zooxanthellae being actively expelled by corals when seawater temperatures increase, to enable re-colonization by different zooxanthellae types that are more efficient at benefiting their host (through photosynthetic carbohydrates) in warmer water^49^. The benefits provided by cnidarian coral hosts to their zooxanthellae symbionts are costly^50^. These include the provision of nitrogen, phosphorous and sulphur nutrients, constant mucus secretion, and producing an often elaborately scleratised morphology that provides the algae shelter whilst simultaneously enabling optimal photosynthesis. Coral bleaching may thus be an extreme manifestation of an adaptive process^49^,^51^ that enables hosts to sanction less-cooperative symbionts, if hosts can be quickly re-colonized with more cooperative zooxanthellae that provide greater net benefits when environmental conditions change. The close spatial endosymbiotic association between partners may thus enable selection for ‘self restraint’ in zooxanthellae in the degree to which they exploit these ‘gifts,’ if this reduces the likelihood of expulsion. ‘Self restraint’ in zooxanthellae may thus contribute to the overall positive correlation we found between host-symbiont reproductive success in these systems. How costs and benefits of cooperation to hosts and symbionts are affected by water temperature is thus a topic of prime importance not only for global conservation, but also to more fully understand variation in reproductive trends in different mutualisms.

Finally, in most mutualisms costs and benefits of cooperation to each mutualist will be asymmetric^7^, and differences in resource use between systems of the host categories used in our study may have contributed to the patterns revealed. Hosts tend to be the ‘dominant’ partner and are thus often able to exert control over their symbionts, for example by partner choice (choosing the most cooperative symbionts) or sanctioning un-cooperative or less cooperative symbionts. Host control may be more profound in cnidarian coral-zooxanthellae mutualisms than the other systems included in our study; the close association between mutualists and the evolution of effective sanctions against potentially less-cooperative symbionts may promote a consistently close association between partner reproductive success. Furthermore, conflict between mutualists may also be more direct in some systems than in others, depending on the nature of the benefits exchanged. In most mutualisms, hosts and symbionts are organisms from different kingdoms that inhabit widely different ecological niches^7^,^34^. The resources exchanged by each partner thus also often differ, e.g. in coral-zooxanthellae mutualisms, reducing the likelihood of overexploitation of hosts by symbionts having a direct negative effect on host reproductive success. However, in some mutualisms, the benefits provided to symbionts directly reduces host reproduction. This is most notable in insect pollination nursery mutualisms, in which the offspring of pollinators are ‘allowed’ to consume some of their plant host’s reproductive tissue in exchange for the pollination services provided by their mothers^52^. When symbionts have the potential to periodically exploit host resources to saturation, their effect on host reproductive success may predictably vary according to the nature of the costs inflicted on the host. We therefore suggest that future quantitative reviews formally account for the exploitative potential symbionts have on their hosts and how this may differ between host taxa.

## Additional Information

**How to cite this article**: Wang, R.-W. *et al.* The importance of spatial heterogeneity and self-restraint on mutualism stability - a quantitative review. *Sci. Rep.* **5**, 14826; doi: 10.1038/srep14826 (2015).

## Supplementary Material

Supplementary Information
