# Supplementary material for: The importance of spatial heterogeneity and self-restraint on mutualism stability - a quantitative review
Source: Sci Rep. 2015 Oct 5;5:14826. doi: 10.1038/srep14826 (PMC4593180; doi:10.1038/srep14826)
Supplement: Supplementary Information [file srep14826-s1.doc]

**The importance of spatial heterogeneity and self****-****restraint on** **mutualism stability - a quantitative review**

Running title: Quantitative review of mutualism maintenance

This is an article

Rui-Wu Wang ^1, 2^ *, Derek W. Dunn ^2^, Jun Luo ^1^, Jun-Zhou He ^2,3^ and Lei Shi ^3*^

1. Center for Ecological and Environmental Sciences, Northwestern Polytechnical University, Xi’an, 710072, China.

2 Kunming Institute of Zoology, Chinese Academy of Science, Kunming, Yunnan, 650223, China. email: [ruiwukiz@hotmail.com](mailto:ruiwukiz@hotmail.com);

3 Statistics and Mathematics College, Yunnan University of Finance and Economics, Kunming, Yunnan, 650221, China.

**Appendix**

**Table A1:** Summary information of the studies used for the meta-analysis

| **Reference** | **No.** | **Symbiont** | **Host** | **Category** | **r** | **N** |
| --- | --- | --- | --- | --- | --- | --- |
| Wang et al. 2011 | 1 | *C. fusciceps* Mayr | *Ficus racemosa* Linn. | woody | 0.74 | 20 |
| Wang et al. 2011 | 2 | *C. fusciceps* Mayr | *Ficus racemosa* Linn. | woody | -0.64 | 21 |
| Wang et al. 2011 | 3 | *C. fusciceps* Mayr | *Ficus racemosa* Linn. | woody | 0.68 | 20 |
| Wang et al. 2011 | 4 | *C. fusciceps* Mayr | *Ficus racemosa* Linn. | woody | -0.07 | 20 |
| Wang et al. 2011 | 5 | *C. fusciceps* Mayr | *Ficus racemosa* Linn. | woody | 0.06 | 20 |
| Wang et al. 2011 | 6 | *C. fusciceps* Mayr | *Ficus racemosa* Linn. | woody | -0.96 | 20 |
| Wang et al. 2011 | 7 | *C. fusciceps* Mayr | *Ficus racemosa* Linn. | woody | 0.75 | 20 |
| Wang et al. 2011 | 8 | *C. fusciceps* Mayr | *Ficus racemosa* Linn. | woody | -0.64 | 20 |
| Zong et al. 2010 | 1 | Eurasuan red squirrel | Arolla pine | woody | 0.87 | 6 |
| Zong et al. 2010 | 2 | Eurasuan red squirrel | Arolla pine | woody | -0.77 | 6 |
| Ando et al. 2011 | 1 | ants | aphids | insect | 0.82 | 20 |
| Ando et al. 2011 | 2 | ants | leaf chewers | insect | -0.75 | 20 |
| Ando et al. 2011 | 3 | ants | sap feeders | insect | -0.77 | 20 |
| Ando et al. 2011 | 4 | ants | leaf chewers | insect | -0.41 | 20 |
| Ando et al. 2011 | 5 | ants | sap feeders | insect | -0.53 | 20 |
| Heath 2010 | 1 | Sinorhizobium meliloti | *Medicago truncatula* | herb | 0.86 | 241 |
| Heath 2010 | 2 | Sinorhizobium meliloti | *Medicago truncatula* | herb | 0.53 | 145 |
| Johnson and Goulet 2007 | 1 | zooxanthellae | cnidarians | coral | -0.73 | 8 |
| Johnson and Goulet 2007 | 2 | zooxanthellae | cnidarians | coral | 0.66 | 24 |
| Mack and Rudgers 2008 | 1 | fungal endophytes | grass | herb | 0.35 | 139 |
| Mack and Rudgers 2008 | 2 | fungal endophytes | grass | herb | 0.03 | 131 |
| Biani et al. 2009 | 1 | mites | bees | insect | 0.14 | 283 |
| Rios et al. 2008 | 1 | ants | plant | herb | 0.76 | 30 |
| Wallace et al. 2008 | 1 | bees | Myriaceae | herb | 0.75 | 30 |
| Wang et al. 2008 | 1 | fig wasps | fig | woody | 0.74 | 13 |
| Wang et al. 2008 | 2 | fig wasps | fig | woody | 0.09 | 25 |
| Wang et al. 2008 | 3 | fig wasps | fig | woody | -0.75 | 21 |
| Katayama and Suzuki 2003 | 1 | *A. craccivora* | *T.caespitum* | insect | 0.56 | 73 |
| Katayama and Suzuki 2003 | 2 | *Ac. pisum* | *T.caespitum* | insect | 0.23 | 73 |
| Katayama and Suzuki 2003 | 3 | *M. crassicauda* | *T.caespitum* | insect | 0.13 | 73 |
| Katayama and Suzuki 2003 | 4 | *A. craccivora* | *L.niger* | insect | 0.86 | 36 |
| Katayama and Suzuki 2003 | 5 | *L. niger* | *Ac. pisum* | insect | 0.1 | 36 |
| Katayama and Suzuki 2003 | 6 | *L. niger* | *M. crassicauda* | insect | 0.01 | 36 |
| Marr et al. 2001 | 1 | pollinator mass | fruit mass | woody | 0.53 | 161 |
| Marr et al. 2001 | 2 | pollinator mass | fruit mass | woody | 0.53 | 137 |
| Marr et al. 2001 | 3 | pollinator mass | fruit mass | woody | 0.17 | 162 |
| Marr et al. 2001 | 4 | pollinator mass | fruit mass | woody | 0.41 | 139 |
| Marr et al. 2001 | 5 | pollinator mass | fruit mass | woody | 0.42 | 181 |
| Hansen et al. 2006 | 1 | ant | *H. mantegazzianum* | herb | 0.15 | 96 |
| Hansen et al. 2006 | 2 | ant | *A. subterranea* | insect | -0.33 | 96 |
| Kobbi et al. 1996 | 1 | *Parapristina verticillata* | *Ficus microcarpa* L. | woody | 0.6 | 21 |
| Kobbi et al. 1996 | 2 | *O. galili* | *Ficus microcarpa* L. | woody | -0.47 | 21 |
| Althoff et al. 2004 | 1 | zooxanthellae | coral | coral | 0.87 | 420 |
| Althoff et al. 2004 | 2 | zooxanthellae | coral | coral | 0.96 | 143 |
| Althoff et al. 2004 | 3 | zooxanthellae | coral | coral | 0.9 | 97 |
| Althoff et al. 2004 | 4 | zooxanthellae | coral | coral | 0.95 | 36 |
| Pauw and Bond 2011 | 1 | *P. catbolocum* | capsule | herb | -0.72 | 36 |
| Pauw and Bond 2011 | 2 | bees | plant | herb | -0.57 | 14 |
| Pauw and Bond 2011 | 3 | bees | plant | herb | -0.62 | 15 |
| Luo 2009 | 1 | zooxanthellae | coral | coral | 0.858 | 10 |
| Clement et al. 2008 | 1 | ant | plant | herb | 0.46 | 21 |
| Clement et al. 2008 | 2 | ant | plant | herb | 0.42 | 29 |
| Tanaka et al. 2011 | 1 | ant | insect | insect | 0.99 | 187 |
| Tanaka et al. 2011 | 2 | ant | insect | insect | 0.99 | 461 |
| Tanaka et al. 2011 | 3 | ant | insect | insect | 0.92 | 1235 |
| Tanaka et al. 2011 | 4 | ant | insect | insect | 0.17 | 54 |
| Bristow 1991 | 1 | ant | aphids | insect | 0.327 | 37 |
| Bristow 1991 | 2 | ant | aphids | insect | 0.668 | 25 |
| Wang ect.2010 | 1 | fig wasps | fig | woody | -0.07 | 8 |
| Wang ect.2010 | 2 | *C. fusciceps* | fig | woody | 0.84 | 15 |
| Wang ect.2010 | 3 | *A. stratheni* | fig | woody | 0.62 | 14 |
| Wang ect.2010 | 4 | *A. fuca* | fig | woody | 0.7 | 15 |
| Wang ect.2010 | 5 | *A. westwoodi* | fig | woody | 0.64 | 14 |
| Adler and Irwin | 1 | pollinator | *G. sempervirens* | Insect | 0.32 | 57 |
| Blair and Williamson 2008 | 1 | *Macrotera lobata* | *Astrophytum asterias* | herb | 0.94 | 7 |
| Blair and Williamson 2008 | 2 | *Macrotera lobata* | *Astrophytum asterias* | herb | 0.67 | 28 |
| Blair and Williamson 2008 | 3 | *Macrotera lobata* | *Astrophytum asterias* | herb | 0.51 | 28 |
| Offenberg et al. 2004 | 1 | ant | leaf damage | herb | -0.8 | 30 |
| Offenberg et al. 2004 | 2 | ant | mangrove | herb | -0.7 | 18 |
| Fründ et al. 2010 | 1 | bees | flower | herb | 0.49 | 27 |
| Fründ et al. 2010 | 2 | bees | flower | herb | 0.41 | 27 |
| Fründ et al. 2010 | 3 | bees | flower | herb | -0.64 | 27 |
| Katayama and Suzuki 2005 | 1 | ant | aphids | insect | -0.213 | 113 |
| Wang ect. 2008 | 1 | fig wasps | *Ficus microcarpa* L. | woody | -0.2 | 15 |
| Wang et al. 2005 | 1 | fig wasps | *Ficus microcarpa* L. | woody | 0.74 | 32 |
| Wang et al. 2005 | 2 | fig wasps | *Ficus microcarpa* L. | woody | -0.59 | 48 |
| Herre and West 1997 | 1 | fig wasps | *Ficus microcarpa* L. | woody | 0.356 | 16 |
| Herre and West 1997 | 2 | fig wasps | *Ficus microcarpa* L. | woody | 0.39 | 16 |
| Herre and West 1997 | 3 | fig wasps | *Ficus microcarpa* L. | woody | 0.23 | 16 |
| Herre and West 1997 | 4 | fig wasps | *Ficus microcarpa* L. | woody | -0.8 | 16 |
| Ehlers et al. 2012 | 1 | *Sinorhizobium meliloti* | *Medicago truncatula* | herb | 0.02 | 12 |
| Ehlers et al. 2012 | 2 | *Sinorhizobium meliloti* | *Medicago truncatula* | herb | 0.57 | 12 |
| Althoff et al. 2012 | 1 | Yucca moth | yucca | herb | -0.157 | 28 |
| Althoff et al. 2012 | 2 | Yucca moth | yucca | herb | 0.11 | 29 |
| Chamberlain and Rudgers 2012 | 1 | Lepidopterans | Gossypium | herb | 0.67 | 28 |
| Chamberlain and Rudgers 2012 | 2 | Lepidopterans | Gossypium | herb | 0.51 | 28 |
| Kudo and Ida 2013 | 1 | Bombus hypocrita | Corydalis ambigua | woody | -0.58 | 49 |
| Kudo and Ida 2013 | 2 | Bombus hypocrita | Corydalis ambigua | woody | 0.091 | 49 |
| Wang et al. 2015 | 1 | *C. fusciceps* Mayr | *Ficus racemosa* Linn | woody | 0.72 | 18 |
| Wang et al. 2015 | 2 | *C. fusciceps* Mayr | *Ficus racemosa* Linn. | woody | -0.18 | 14 |
| Wang et al. 2015 | 3 | *C. fusciceps* Mayr | *Ficus racemosa* Linn. | woody | -0.65 | 26 |
| Wang et al. 2015 | 4 | *C. fusciceps* Mayr | *Ficus racemosa* Linn | woody | 0.74 | 23 |
| Wang et al. 2015 | 5 | *C. fusciceps* Mayr | *Ficus racemosa* Linn. | woody | -0.79 | 21 |
| Wang et al. 2015 | 6 | *C. fusciceps* Mayr | *Ficus racemosa* Linn. | woody | 0.61 | 41 |
| Wang et al. 2015 | 7 | *C. fusciceps* Mayr | *Ficus racemosa* Linn | woody | -0.51 | 24 |
| Vega and Herrera 2012 | 1 | *Metschnikowia reukaufii* | *Cytinus hypocistis* | woody | -0.629 | 15 |

In inter-specific mutualisms, cheats are individuals within a population (usually symbionts) that benefit from the cooperative actions of other symbionts and the host, but in return provide the host no or reduced benefits[^1^](#_ENREF_1). Cheats thus incur no or reduced costs of cooperation, whilst still receiving benefits associated with host resource exploitation[^1^](#_ENREF_1). If host resources become fully exploited, cooperative symbionts will thus subsequently receive no benefits even though they incur costs of cooperation[^2-4^](#_ENREF_2).

Any spatial heterogeneity of hosts or host resources may not be able to solely stabilise a mutualism. Spatial heterogeneity may often evolve but can ultimately fail to result in the evolution of cooperation, because a mutant able to overcome spatial heterogeneity barriers to exploit the other mutualist will have higher short-term fitness than cooperative conspecifics in a limited local space[^5^](#_ENREF_5). In some mutualisms, it has been shown empirically that spatial heterogeneity or self restraint may be unable to solely maintain system stability (e.g., in the fig tree-fig wasp mutualism[^6-8^](#_ENREF_6), such as the mutualism between the moth *Greya politella* and its host plant *Lithophragma parviflorum*[^9^](#_ENREF_9).

**References**

1 Ghoul, M., Griffin, A. S. & West, S. A. Toward an evolutionary definition of cheating. *Evolution* **68**, 318-331 (2014)

2 Doebeli, M. & Knowlton, N. The evolution of interspecific mutualisms. *Proc. Nati. Acad. Sci. USA* **95**, 8676-8680 (1998).

3 Boyd, R. & Lorberbaum, J. P. No Pure Strategy Is Evolutionarily Stable in the Repeated Prisoners-Dilemma Game. *Nature* **327**, 58-59 (1987).

4 Wang, R. W. & Shi, L. The evolution of cooperation in asymmetric systems. *Sci. China Life Sci.* **53**, 139-149 (2010).

5 Hauert, C. & Doebeli, M. Spatial structure often inhibits the evolution of cooperation in the snowdrift game. *Nature* **428**, 643-646 (2004).

6 Dunn, D. W. *et al.* A Role for Parasites in Stabilising the Fig-Pollinator Mutualism. *PloS Biol.* **6**, e59 (2008).

7 Wang, R. W., Shi, L., Ai, S. M. & Zheng, Q. Trade-off between reciprocal mutualists: local resource availability-oriented interaction in fig/fig wasp mutualism. *J. Anim. Ecol.* **77**, 616-623 (2008).

8 Wang, R. W., Sun, B. F. & Zheng, Q. Diffusive coevolution and mutualism maintenance mechanisms in a fig-fig wasp system. *Ecology* **91**, 1308-1316 (2010).

9 Thompson, J. N. & Fernandez, C. C. Temporal dynamics of antagonism and mutualism in a geographically variable plant-insect interaction. *Ecology* **87**, 103-112 (2006).
